# Virologic, immunologic and clinical response of infants to antiretroviral therapy in Kampala, Uganda

**DOI:** 10.1186/1471-2431-13-42

**Published:** 2013-03-27

**Authors:** Vincent J Tukei, Miriam Murungi, Alice R Asiimwe, Daniella Migisha, Albert Maganda, Sabrina Bakeera-Kitaka, Israel Kalyesubula, Philippa Musoke, Adeodata Kekitiinwa

**Affiliations:** 1 Baylor College of Medicine-Bristol Myers Squibb Children’s Clinical Center of Excellence at Mulago Hospital, Kampala, Uganda; 2 Makerere University-Johns Hopkins University Research Collaboration Clinic, Kampala, Uganda; 3 Department of Pediatrics and Child Health, Makerere University College of Health Sciences, Kampala, Uganda

**Keywords:** Infant, HIV, Antiretroviral therapy, Mortality, Malnutrition

## Abstract

**Background:**

Antiretroviral therapy (ART) is known to save lives. Among HIV-infected infants living in resource constrained settings, the short and long term benefits of ART are only partially known. This study was designed to determine the virologic, immunologic and clinical outcomes of antiretroviral therapy in a cohort of HIV-infected infants receiving care from an outpatient clinic in Kampala, Uganda.

**Methods:**

A prospective cohort of HIV-infected infants receiving treatment at the Baylor-Uganda clinic was analyzed. Patients were diagnosed, enrolled and followed up at the clinic. HIV viral load, CD4 cell counts and clinical progress were assessed during follow-up. Descriptive statistical analysis and logistic regression modeling to determine predictors of treatment success were conducted.

**Results:**

Of 91 HIV-infected infants enrolled into the cohort, 53 (58.2%) infants were female; 43 (47.3%) were 6 months of age or younger, and 50 (55.6%) had advanced HIV/AIDS disease (Clinical stage 3 or 4). Eighty four infants started ART and 78 (92.9%) completed 6 months of treatments. Fifty six (71.8%) infants attained virologic suppression by month-6 of ART, and at month-12 of ART, the cumulative probability of attaining viral suppression was 83.1%. None of the baseline infant factors (age, sex, WHO stage, CD4 cell percent, weight for age, or height for age z-score) predicted treatment success. There was an increase in CD4 cells from a baseline mean of 23% to 30% at month-6 of treatment (p<0.001) and by month-24 of ART, the mean CD4 percent was 36%. A total of 7 patients died while on ART and another 7 experienced adverse events that were related to treatment.

**Conclusion:**

Our results show that, even among very young patients from resource constrained settings, ART dramatically suppresses HIV replication, allows immune recovery and clinical improvement, and is safe. However, baseline characteristics do not predict recovery in this age group.

## Background

Over the last decade, the beneficial effects of antiretroviral therapy (ART) have been reported among HIV-infected adults and children in both resource rich and resource poor countries [1-4]. Antiretroviral therapy markedly reduces morbidity and mortality by suppressing viral replication which ultimately leads to immune recovery and a reduction in opportunistic infections. Among infants and children, early introduction of ART is known to save lives [5-7]. However, the effects of early initiation of life-long treatment among infants receiving care in HIV treatment programs in the resource constrained settings of Africa are not fully known.

The World Health Organization (WHO) in its 2010 revision of HIV treatment guidelines recommends early initiation of ART among infants and young children [8]. As these recommendations are being implemented across Africa, a sizable number of HIV-infected infants, some as young as 6 weeks, are being started on these daily medications. The short and long term effects of early ART initiation among these very young patients are yet to be fully known. The frequency of adverse events among these infants, and the level of immunologic and clinical recovery that occurs when these drugs are administered to infants outside of the controlled environment of clinical trials are yet to be determined.

The objective of this study was to determine the virologic response to ART in a cohort of HIV-infected Ugandan infants (children less than 12 months of age) as measured by HIV viral suppression after 6 months of therapy; determine the level of immunologic recovery as measured by increase in CD4 cell counts; and determine the proportion of infants developing ART-related adverse events.

## Methods

### Study design and setting

For this observational cohort study, we recruited and followed HIV-infected infants who were enrolling for care and treatment at the Baylor College of Medicine Bristol Myers Squibb Children’s clinical centre of excellence at Mulago Hospital (Baylor-Uganda) in Kampala, Uganda. Baylor-Uganda is a Non Governmental Organization (NGO) involved in the prevention, care and treatment of HIV-infected children and their families in Uganda. The Foundation is donor funded and runs a clinic that offers free clinical care to all its patients. The clinic is an out-patient treatment center that operates five days a week with an average daily attendance of 170 patients. Over 90% of patients seen at the clinic are children from the age of 6 weeks to 18 years. Parents or adult caretakers constitute the remainder of the patients.

At the clinic, patient care involves HIV diagnosis and treatment, nutritional rehabilitation, diagnosis and treatment of opportunistic infections, counseling and social support. Infants enrolled for care at the clinic are referred from the nearby Mulago National Referral hospital wards and from other centers involved in Prevention of Mother to Child HIV Transmission (PMTCT). Some infants are referred from HIV treatment centers within the country, and others are self referrals.

### Study participants

Study participants were identified and recruited into the cohort from the Baylor-Uganda clinic patient population. Study participants were selected based on a criteria that required them to be clinic patients, confirmed to be HIV positive (by 2 positive blood tests showing the presence of viral nucleic acids in the patient’s blood samples); between 6-weeks and 12 months of age; ART naïve, but willing to start ART as soon as deemed necessary by the clinician; reside within 20 kilometer radius from the clinic; willing to be visited at home; and agree to be regularly followed-up at the clinic. Children without parents or guardians were excluded. Patients less than 6 weeks of age were not included since they were not routinely registered for care at the clinic. Based on WHO guidelines for infant diagnosis [9], and recommendations from the Ugandan Ministry of Health, routine diagnosis of HIV at Baylor-Uganda is carried out from the age of 6 weeks when infants are brought back to hospital for immunization.

From April 2009 to March 2012, all clinic patients that fulfilled the selection criteria were consecutively recruited into the cohort and were subsequently followed up.

### Follow-up procedures and variables measured

Before starting ART, we confirmed HIV infection in the infants by carrying out an HIV DNA Polymerase Chain Reaction (PCR) test followed 2 weeks later by a viral RNA PCR test to quantify the HIV virus in the blood. To be considered HIV infected, both tests had to confirm presence of the virus in blood.

All laboratory- confirmed HIV infected infants were started on ART. Infants enrolled before July 2010 were started on ART based on the 2006 WHO guidelines for ART initiation. Based on these guidelines, all infants with CD4 cell counts below 25% or in advanced WHO clinical stages (stages 3 and 4) were considered eligible for ART and were started on treatment. Following the 2010 revision of WHO treatment guidelines, all HIV-infected infants were started on ART irrespective of their clinical stage or CD4 cell counts. A combination of 2 Nucleoside Reverse transcriptase inhibitor (NRTI) drugs and 1 Non-Nucleoside Reverse Transcriptase (NNRTI) or Protease Inhibitor was the ART combination of choice. Zidovudine (ZDV) or stavudine (D4T) combined with lamivudine (3TC) together with either nevirapine (NVP) or Lopinavir/ritonavir were the main ART drug combinations as recommended by WHO. Stavudine was preferred in patients with anemia (hemoglobin <7.5 g/dl) and Lopinavir/ritonavir was preferred in patients exposed to single dose NVP for PMTCT. Patients on rifampicin for treatment of tuberculosis were put on 3 NRTIs (ZDV or D4T + 3TC + Abacavir).

Baseline measurements and tests were carried out on the date when ART was started and subsequent follow-up visits were scheduled 2, 4, and 8 weeks after starting treatment. Thereafter, patients returned to the clinic monthly, and if an appointment was missed, a home visit was carried out by trained home health workers. In addition, parents were encouraged to bring the children for medical care whenever they fell sick. At baseline and every 6 months thereafter, blood samples were drawn for complete blood count (CBC), CD4 cell count; liver function test [serum alanine amino transferase (ALT)], and HIV viral load.

At every visit, physicians reviewed the infant, documented and managed any adverse events or opportunistic infections. In addition, nutritional assessment including measurement of weight and height was conducted. Anthropometric computations and comparisons were conducted using the WHO anthro software [10] which is based on WHO child growth standards. Underweight was defined as a weight for age z-score (WAZ-score) less than -2 standard deviations (SD) from the WHO reference median values. Stunting was defined as height for age z-score (HAZ-score) less than -2SD from the reference values and weight for height z-score (WHZ-score) less than -2SD from the WHO reference median defined wasting. Adherence to medication was encouraged by both the physician and a trained counselor.

Antiretroviral therapy was considered the exposure variable and the primary outcome variable was viral suppression 6 months after ART initiation. Other outcomes of interest included change in CD4 cell count; occurrence of ART-related adverse events; death during follow-up; and change in nutritional status as measured by weight-for-age, height-for-age and weight for height z-scores. Virologic suppression was defined as the attainment of HIV viral loads less than 400 copies/ml after 6 months of ART.

Patients’ age, sex, WHO clinical stage, ART combination, presence of opportunistic infections and concurrent use of other medication, were considered potential confounders and were therefore documented and considered in the analysis.

All patient information was recorded in structured forms and was subsequently entered into the clinics electronic medical database which is designed to check for consistency of the data.

Written approval to conduct the study was granted by Makerere University College of Health Sciences’ Research and Ethics committee, Uganda National Council of Science and Technology, and Baylor College of Medicine Institutional Review Board (USA). The parents or caretakers gave written informed consent to participate in the study.

### Statistical analysis

Descriptive analyses of baseline and follow-up characteristics are presented as proportions, medians with inter-quartile ranges (IQR), and means with standard deviations (SD).We used the paired *t*-test to compare the mean CD4 cell counts at baseline and during follow-up. Virologic suppression was categorized as ‘successful’ for viral loads less than 400 copies per milliliter and as ‘unsuccessful’ if otherwise. We used bivariate and multivariable logistic regression models to identify predictors of successful virologic suppression. Proportions of infants developing ART related adverse events were computed. For all analyses, confidence intervals (CI) were set at the 95% level and p-values less than 0.05 were considered statistically significant.

## Results

Of 91 HIV-infected infants enrolled into the cohort during the study period, 53(58.2%) patients were female; 43(47.3%) were 6 months of age or younger, and 57 (62.7%) were moderate or severely underweight (Table 1). The median time from initial registration at the clinic to ART initiation was 57 days (IQR: 34.5-89). This duration varied according to ART initiation guidelines used at the clinic. The median time to ART initiation for patients that were started based on 2006 WHO treatment guidelines was 69 days (IQR=47-115); whereas the duration to ART initiation for patients that were started based on the revised 2010 guidelines was 33 days(IQR=28-44). The median follow-up time after initiating ART was 752 days (IQR= 531-980). The median age at ART initiation was 28.3 weeks (IQR=19.7-39) for infants started under the 2006 guidelines and 22.6 weeks (IQR=17.1-34.7) for infants that were started under 2010 WHO treatment guidelines.

**Table 1:** Baseline characteristics of a cohort of 91 HIV-infected infants followed up at Baylor-Uganda

**Variable**	**Median (IQR)**	**N (%)**
Age (months)	6.2 (4.04-9.0)	
0-6		43 (47.3)
7-12		48 (52.7)
Sex		
Male		38 (41.8)
Female		53 (58.2)
CD4 Percent	20 (15-29)	
Viral load (copies/ml)		
<100,000		14 (18.2)
100,000-750,000		29 (37.7)
>750,000		34 (44.1)
WHO clinical stage		
1		13 (14.4)
2		27 (30.0)
3		32 (35.6)
4		18 (20.0)
ART regimen		
ZDV+3TC+NVP		14 (16.7)
ZDV+3TC+Lop/r		13 (15.5)
D4T+3TC+NVP		30 (35.7)
D4T+3TC+Lop/r		16 (19.0)
Other		11 (13.1)
Weight/Age z-score		
Median (IQR)	−2.2 (-3.89 - -1)	
<-3SD score		33 (36.3)
≥-3SD to <-2SD		24 (26.4)
≥-2≤SD to < -1SD		11 (12.1)
≥-1SD		23 (25.2)
Height/Age z-score		
Median	−1.92 (-3.05 - -1.02)	
<-3SD score		23 (25.3)
≥-3SD to<-2SD		20 (21.9)
≥-2SD to<-1SD		26 (28.6)
≥-1SD		22 (24.2)

IQR= Inter-quartile range, SD= standard deviation, WHO= World Health Organization, ART=antiretroviral therapy, ZDV=zidovudine, 3TC=lamivudine, D4T=stavudine, NVP=nevirapine, Lop/r= Lopinavir/ritonavir.

During the follow-up period, a total of 84 patients were started on ART; 59 (70.2%) infants were started based on the 2006 WHO treatment guidelines and 25(29.8%) were started based on the revised 2010 treatment guidelines. Seven infants were not started on treatment because 4 of them died shortly after enrolment and 3 were lost to follow-up before ART could be initiated. Among infants that started ART, 78 (92.9%) were in active care at the end of the first 6 months of therapy; 4 patients died and 2 were lost to follow-up within the 6 month period. At the end of the study period, 70, 62 and 47 patients had received ART for at least 12, 18 and 24 months respectively.

### Virologic response

Of the 78 infants that had ART for at least 6 months, successful viral suppression was seen in 56(71.8%) infants. Twenty two (28.2%) infants continued to have detectable viremia at month-6 of treatment. In univariate and multivariable regression analysis; none of the baseline factors such as age, gender, CD4 cell percent, WHO clinical stage, choice of antiretroviral agent, weight for age or height for age z-scores could predict treatment success at month-6 of therapy (Table 2).

**Table 2:** Baseline predictors of treatment success (viral load<400 copies) among HIV-infected infants 6 months after ART initiation

**Characteristic**	**Univariate OR**	**P-value**	**Multivariable OR**	**P-value**
	**(95% CI)**		**(95% CI)**	
Age (>6 months)	0.83 (0.31-2.24)	0.718	1.20 (0.33-4.37)	0.785
Sex (Male)	1.01 (0.37-2.74)	0.990	0.77 (0.24-2.45)	0.657
CD4 percent (every 1%)	0.99 (0.94-1.04)	0.670	0.95 (0.89-1.01)	0.123
WHO stage (stage3&4)	0.43 (0.15-1.23)	0.116	0.33 (0.08-1.14)	0.078
Protease Inhibitor	1.34 (0.45-4.02)	0.603	1.33 (038-4.65)	0.657
Weight for Age Z-score (<-2)	0.63 (0.21-1.85)	0.395	0.40 (0.09-1.76)	0.227
Height for age Z-score (<-2)	1.44 (0.50-4.12)	0.500	2.08 (0.58-7.47)	0.260

ART= antiretroviral therapy; OR= Odds Ratio; CI= Confidence Interval; WHO= World Health Organization.

At month-12 of ART, 8 of the 22 patients that had detectable viremia at month-6, attained viral suppression; 1 patient got lost to follow-up, 1 was switched to second line ART, and 12 patients continued to have detectable viremia. On subsequent clinic visits, 6 of the 12 patients were switched to second-line ART, 4 were lost to follow-up and 2 died (Table 3). Among patients that attained viral suppression at month-6, no viral rebound was seen at month-12 of ART.

**Table 3:** Characteristics of 12 children with virologic treatment failure after 12 months of antiretroviral therapy

**Serial number**	**Age at ART initiation(months)**	**WHO clinical stage**	**ART regimen**	**Other significant OIs/social issues**	**Outcome**
1	4	2	ZDV-3TC-NVP	None	Lost to follow-up
2	4	3	D4T-3TC-LPV/r	None	Switched to second line ART
3	5	3	ZDV-3TC-NVP	3 Pneumonia episodes	Switched to second line ART
4	7	3	D4T-3TC-ABC	Tuberculosis	Died
5	11	3	ZDV-3TC-ABC	Tuberculosis	Lost to follow-up
6	6	3	D4T-3TC-NVP	Chronic suppurative otitis media	Switched to second line ART
7	6	3	D4T-3TC-NVP	None	Lost to follow-up
8	4	2	ZDV-3TC-NVP	None	Switched to second line ART
9	10	3	D4T-3TC-NVP	Poor adherence to ART	Switched to second line ART
10	10	2	D4T-3TC-LPV/r	Teenage Mother	Lost to follow-up
11	8	3	D4T-3TC-NVP	None	Switched to second line ART
12	10	4	ZDV-3TC-NVP	Anemia probably due to ZDV	Died

WHO= World Health Organization; ART= Antiretroviral therapy; OI= opportunistic infections.

Of 47 patients that completed 24 months of ART; 44(93.6%) were still on first line treatment. Of these, 37(84.1%) continued to have viral suppression; and 7 patients had detectable viremia, all of whom had earlier achieved viral suppression.

Altogether 7 patients were switched to second-line treatment (Abacavir + 3TC + Lopinavir/ritonavir) as a result of persistent viremia;5 out of the 7 attained viral suppression 6 months after starting second-line ART, and 2 patients were yet to complete 6 months of second-line ART.

The cumulative probabilities of attaining viral suppression at month-6 and month-12 of ART were 71.8% (95% CI: 61.6-81.3) and 83.1% (95% CI: 73.8-90.5) respectively.

The choice of third agent (Nevirapine versus Lopinavir/r) was not associated with risk of viremia at month-6, even after adjusting for the choice of NRTI backbone and baseline WHO clinical stage, Risk Ratio(RR)=1.1 (95% CI: 0.8-1.5), P=0.152. There was no association between WHO guideline used for patient treatment (2006 versus 2010) and risk of virologic failure at month-6 of ART; RR=1.24 (95% CI: 0.9-1.6).

### Immunologic response

**Figure 1:**
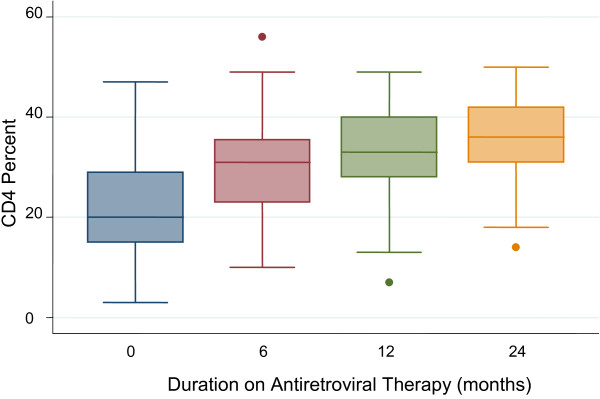
Change in CD4 cell counts in a cohort of children receiving antiretroviral therapy at Baylor-Uganda.

There was a significant increase in CD4 cells from a baseline mean of 23% (SD=10.1) to 30% (SD=9.6) at month-6, P<0.001; and to 33% (SD=8.9) at month-12 of ART; (P=0.009, for the change between month-6 and month-12). By the end of the second year of ART, the mean CD4 cell percentage rose to 36% (SD=8.05) (Figure 1). The proportion of infants with very low CD4 cell counts (<15%) dropped from 23.8% at baseline to 8.5% at 6 months of treatment, and by the end of the second year, only one patient continued to have CD4 cell percentage below 15%. This patient was an infant, with no prior exposure to nevirapine for PMTCT, who started ART (D4T+3TC+NVP) at the age of 4 months with a baseline CD4 of 16%. His CD4 percentage dropped to 15% at month-6 and to 14% at month-24 of ART. He never attained virologic suppression and was considered a case of treatment failure due to possible primary HIV resistance. He was subsequently started on second-line ART.

### Clinical response

Ten (11.9%) of the 84 patients that started ART were diagnosed with tuberculosis at baseline or shortly after ART initiation. Anti-tuberculosis treatment was started but one patient died after 5 months of ART and tuberculosis medication. One 7-month old infant was diagnosed with disseminated Bacillus Calmette-Guerin (BCG) infection 3 months after starting ART. He was treated as a case of immune reconstitution inflammatory syndrome (IRIS) and improved.

At ART initiation, 54 (64.3%) of 84 patients had a WAZ-score below -2SD of the WHO reference median. This prevalence of underweight varied according to age and clinical stage of the disease. Thirty six (80%) of 45 older infants (7-12 months of age) compared to 18(46.2%) of 39 younger infants (1-6 months of age) were underweight at baseline; prevalence ratio= 1.7 (95% CI: 1.19-2.51). Of 47 infants with baseline advanced HIV disease (WHO clinical stage 3&4), 36 (76.6%) were underweight; and among 37 infants with milder clinical disease (WHO clinical stage 1&2), 18 (48.6%) were found to be underweight at baseline; prevalence ratio= 1.6 (95% CI: 1.09-2.27). There was no association between baseline underweight and gender or baseline CD4 cell counts.

Among the 78 infants that received ART for at least 6 months, the proportion that were underweight reduced from 65.4% at baseline to 30.8% at 6 month of ART, p<0.001

Eighty one patients were included in the analysis of height for age z-scores (HAZ-score). Of these, 32 (39.5%) patients had baseline HAZ-score less than -2. Twenty (46.5%) out of 43 infants aged 6-12 months compared to 12 (31.5%) out of 38 infants younger than 6 months of age were stunted; prevalence ratio=1.5 (95% CI: 0.84-2.60). Twenty three (51.1%) of 45 patients in WHO stage 3or 4 compared to 9 (25%) of 36 patients in WHO stage 1 or 2 were stunted at baseline; prevalence ratio=2 (95% CI: 1.08-3.85).

There was no significant change in stunting in the first 6 month of ART. Of 75 patients that had both baseline and follow-up HAZ-score measurements, the prevalence of stunting increased from 40% at baseline to 48% after 6 months of ART, p=0.134.

Of 65 patients that were included in the analysis of weight for height z-scores (WHZ-score), 22 (33.8%) had baseline WHZ-scores less than -2. However, after 6 months of ART, only 9 (13.8%) patients continued to have WHZ-scores less than -2; p=0.007.

### Deaths and other adverse events

A total of 11 (12.1%) patients died during the follow-up period. Four of the 11 died before ART could be initiated. All 4 patients were in WHO clinical stage 4; between 6-12 months of age at the time of death; and had been in care for 6-11.7 weeks. The illness preceding death was tuberculous pericarditis for one female infant, dysentery and lymphoma for other 2 female infants and Pneumocystis Jirovecii Pneumonia for the only male infant. Of the 7 infants that died while on ART, 4 died within the first 6 months, 1 died in the 8^th^ month and 2 died in the second year of ART. The overall mortality rate among children on ART was 4.09 (95% CI: 1.95-8.58) per 100 child-years. Post mortem examinations were not carried out; however, the majority of patients that died had underlying malnutrition (Table 4).

**Table 4:** Characteristics of 7 HIV-infected Children that died while on antiretroviral therapy at Baylor-Uganda clinic

**Serial number**	**Age(months) at ART initiation**	**Sex**	**WHO clinical stage**	**ART combination**	**Duration on ART (months)**	**Clinical events preceding death**
1	7	Male	3	D4T-3TC-ABC	18.3	Tuberculosis
2	7	Female	4	D4T-3TC-NVP	16.3	Malnutrition
3	6	Female	3	D4T-3TC-NVP	7.8	Pneumonia
4	7	Male	4	D4T-3TC-NVP	0.8	Malnutrition/Anemia
5	11	Male	4	D4T-3TC-NVP	1.3	Malnutrition
6	7	Female	3	D4T-3TC-NVP	1.7	Oral Candidiasis
7	10	Male	4	D4T-3TC-NVP	5.3	Malnutrition

ART= Antiretroviral therapy; WHO= World Health Organization; D4T= Stavudine; 3TC= Lamivudine; ABC= Abacavir; NVP= Nevirapine.

Within the first 6 months of ART, adverse events were reported in 7 (8.3%) of the 84 patients that started treatment. Of the seven, 3 patients developed diarrhea, 1 had nausea and vomiting, 2 had anemia and 1 developed a skin rash. One of the 3 infants with diarrhea and the one infant with nausea and vomiting were on a PI based regimen. All patients with anemia were on ZDV and the one patient with skin rash was on nevirapine. All patients that developed anemia or skin rash had ART regimen change. Patients with diarrhea, nausea and vomiting had mild and transient illness not requiring ART regimen change.

## Discussion

In this study, which involved a cohort of HIV-infected infants receiving first-line antiretroviral therapy in an outpatient setting in Uganda, we found that 71% of infants attained virologic suppression by 6 months of ART. In general, the infants had good immune recovery and experienced low levels of drug toxicity, with only 8% of patients reporting adverse events during the study period. Our results show that, even among very young infants from resource constrained settings, ART dramatically suppresses HIV replication, allows immune recovery and clinical improvement, and is safe.

In sub-Saharan Africa, the treatment of HIV-infected infants has lagged behind that of older children and adults largely as a result of delays in diagnosis [11], restricted choice of ART, lack of appropriate drug formulations and inadequate levels of trained medical personnel [12,13]. These difficulties coupled with the high rates of viral resistance among infants that received nevirapine for PMTCT, complicate the attainment of treatment success [14,15]. However, despite these challenges, the majority of infants in our cohort were adequately able to suppress the virus, increase their level of immunity and significantly gain weight. Our findings on growth recovery provide further evidence on the benefits of early initiation of ART. Recent reports by Shiau et al suggest that growth recovery is faster among young infants compared to older children [16].

Compared to participants of the CHER study [7], infants in our cohort were enrolled into care at an older age, and were started on ART after several weeks of delay. This may have partly contributed to the mortality and loss to follow up seen before ART initiation. The 2010 revision of WHO treatment guidelines led to a notable reduction in ART initiation delay, with much younger infants being enrolled into care. The implementation of the 2010 treatment guidelines together with the increased adoption of strategies for early infant diagnosis (EID) across Uganda and other parts of Africa, are likely to lead to further reduction in ART initiation delays and early enrollment of infants into care.

Among several cohorts of HIV-infected African children currently undergoing treatment, viral suppression within the first 6 months of ART has been reported in 60% to 89% of patients [17-21]. Our results, though purely from infants, are in agreement with results from these studies. Studies conducted in Europe and primarily focusing on infants have reported much lower responses to therapy [22].

In all patients on ART, the initial rapid attainment of viral suppression is critical for the long term success of treatment. Viral suppression permits immune recovery and often delays the development of viral resistance [20]. Persistence of HIV viremia among patients on ART leads to early development of viral resistance ultimately leading to treatment failure. With the achievement of viral suppression, immune recovery and clinical improvement often occur. However, attainment of HIV viral suppression and immune rebound is often dependent on several factors including; the choice of ART regimen, prior treatment or exposure to antiretroviral drugs for PMTCT, adherence to medication, drug dosage, concurrent use of other drugs, presence of other infections and a host of other factors [23-25]. Our study did not assess all these factors; however, among baseline variables measured, we did not find any associations between infant characteristics and viral suppression. Neither was there any association between viral suppression and ART regimen used. Viral suppression was seen irrespective of age, sex, WHO clinical stage or baseline CD4 cell counts.

The immune recovery that occurred in our cohort was remarkable. The change from a baseline mean of 22% to 30% after 6 months of ART compares favorably with results seen among cohorts of children receiving treatment elsewhere in Africa [5]. In general, the increase in CD4 cell count continued even during the second year of therapy.

Despite this general success in treatment, several children failed to attain viral suppression, even after 6-12 month of first-line treatment. We presume that these children may have been infected with mutant resistant viruses from mothers that were already on treatment with first-line ART. Though HIV resistance tests were not carried out, our conclusion is that these children may have been cases of primary HIV resistance, or cases of resistance resulting from prior use of antiretroviral agents for PMTCT. Once switched to second line ART, viral suppression was quickly attained. These results are worrying since it implies that either the mothers on treatment are passing on resistant virus to their infants, or prior administration of PMTCT medication to infants maybe contributing to the development of viral resistance. This development further limits the treatment options available for these children. With more adults accessing and failing first-line ART, these results underscore the need for HIV resistance testing among HIV-infected infants born to such adults.

The viral rebound that occurred in 7 patients after initial suppression may have been due to early viral resistance or due to poor adherence to medication at the time the viral load tests were carried out.

We noted a high level of acute and chronic malnutrition in this cohort and this may have been due to a combination of clinical and social factors that ultimately led to reduced caloric intake, reduced absorption and increased loss of nutrients needed for growth. This high prevalence of malnutrition underscores the need for an integrated HIV treatment approach that incorporates interventions focusing on reducing malnutrition.

As reported in previous studies [21,26], deaths were mainly reported in the first 6 months of ART. Early deaths in HIV-infected children are often a result of overwhelming infections in a setting of severe immunodeficiency and malnutrition [27,28]. Due to the rapid progression of disease and the delays in diagnosis, many infants enroll for medical care when infection, malnutrition and severe immunodeficiency have set in. For such patients, death occurs before the beneficial effects of ART are realized. Mortality rates reported from several African cohorts vary considerably; in 2 South African cohorts, rates of 4.2 deaths per 100 child years [21] and 43.7 deaths per 1000 child-years were reported [5]. In Kenya, 8.4 deaths/100 child-years were seen [29] and in Zambia 6.94 deaths per 100 person years were reported [30]. The mortality rate of 4.09 deaths per 100 child-years seen in our cohort compares favorably with these results.

Our study had several limitations: We were not able to accurately document PMTCT history; neither were we able to carry out HIV resistance testing. In addition, we were unable to carry out postmortem examination of patients that died, and we did not measure adherence to medication though it was emphasized at every clinic visit. Despite these limitations and the possibility of failing to detect significant differences due to the small sample size, the results presented here draw attention to a vulnerable group of HIV-infected children and highlight the feasibility of treatment success even in resource constraint settings.

## Conclusion

Our results indicate that despite the high prevalence of malnutrition and the probable transmission of resistant virus from mothers to infants, treatment success for HIV-infected infants living in resource-constrained settings is possible. We recommend an integrated approach to early infant diagnosis and treatment that takes into consideration early detection of viral resistance especially among infants whose mothers received ART during pregnancy and a robust nutritional intervention plan for every infant.

## Competing interests

The authors declare that they have no competing interests.

## Authors’ contributions

VJT participated in the design of the study, acquisition of data, data cleaning and analysis, and drafting, revision and final submission of the manuscript. MM participated is data acquisition and in the critical revision of the manuscript. ARA was involved in study design, submissions for ethical approval and critical revision of the draft manuscript. DM participated in the design of the study and acquisition of data. AM was involved in the initial design of the study and critical revision of the draft manuscript. SBK, IK and PM all participated in the initial design of the study, interpretation of the data and critical revision of the draft manuscript. AK participated in all stages of the study from design, data acquisition, interpretation of collected data, and final revision of the draft manuscript. All authors read and approved the final manuscript.

## Pre-publication history

The pre-publication history for this paper can be accessed here:

http://www.biomedcentral.com/1471-2431/13/42/prepub
